# Laparoscopic surgery for De Garengeot’s hernia in a man after inguinal hernia surgery with a mesh plug: a case report and review of literature

**DOI:** 10.1186/s40792-024-01925-7

**Published:** 2024-05-29

**Authors:** Shiro Fujihata, Hiromasa Kuzuya, Masaaki Kurimoto, Tadashi Shibata, Hirozumi Sawai, Shuji Takiguchi

**Affiliations:** 1 Department of Surgery, Narita Memorial Hospital, 134 Hanei-Honmchi, Toyohashi, Aichi Japan; 2 Department of Gastroenterological Surgery, Nagoya City University Graduate School of Medical Sciences, Mizuho-Cho, 1 Kawasumi, Mizuho-Ku, Nagoya, Aichi Japan

**Keywords:** De Garengeot’s hernia, Laparoscopic surgery, Transabdominal preperitoneal approach (TAPP), Recurrent groin hernia

## Abstract

**Background:**

De Garengeot’s hernia is a rare case of a femoral hernia that contains the appendix. Here we report a case of De Garengeot’s hernia that occurred in a male patient who had a history of inguinal hernia surgery using a mesh plug. There were no reports of De Garengeot’s hernia with a history of surgery for inguinal hernia, and the surgical question was whether we could successfully treat a patient with minimally invasive laparoscopic surgery using a mesh.

**Case presentation:**

This case involved 75-year-old man with a history of right indirect inguinal hernia surgery using a mesh plug without on-lay mesh, who presented with a 5-day history of a right groin lump. Abdominal CT revealed an incarcerated appendix within the right femoral hernia and fluid collection around the appendix. Laparoscopic surgery was initiated and the incarcerated appendix was released with traction. There was no contamination around the appendix or the femoral ring, the appendix was removed, and the femoral hernia was repaired using mesh. Laparoscopic surgery was useful in first evaluating the inflammatory status of the appendix. As it was determined that there was little inflammation around the appendix and femoral ring, it was possible to repair the hernia using mesh.

**Conclusions:**

De Garengeot’s hernias are rare and there is currently no standardized approach. Even if it is a recurrent hernia in the groin, laparoscopic surgery can be useful for diagnosis and treatment, but the use of mesh requires further careful consideration.

## Background

De Garengeot’s hernia is a rare subtype of a femoral hernia that contains the appendix. According to the Swedish Hernia Register [1], femoral hernias accounted for 2.8% of 141,916 patients, 67% of whom were women. The frequency of De Garengeot’s hernias among all femoral hernias is thought to be around 1%, but past data have been cited without being updated for a long time, and more accurate data in recent years are unknown [2]. Similar to Amyand’s hernia, in which the content of the inguinal hernia is the appendix, a De Garengeot’s hernia can cause inflammation and necrosis of the appendix, requiring emergency surgery. Due to the small number of cases of these groin hernias involving the appendix, there is still no consensus regarding the appropriateness of using mesh or laparoscopy for treatment. Here we report a case of De Garengeot’s hernia that occurred in a male patient who had a history of inguinal hernia surgery using a mesh plug. There were no reports of De Garengeot’s hernia with a history of surgery for inguinal hernia, and we successfully treated the patient with minimally invasive laparoscopic surgery using a mesh.

## Case presentation

A 75-year-old man, with a history of mesh plug repair for a right indirect inguinal hernia 8 years ago, presented to the emergency department of a secondary care hospital with a right groin lump. He had felt a lump in his right groin for 5 days, but there was no pain. His body mass index (BMI) was 19.57 kg/m^2^, and he was afebrile and had normal pulse and blood pressure.

**Fig. 1:**
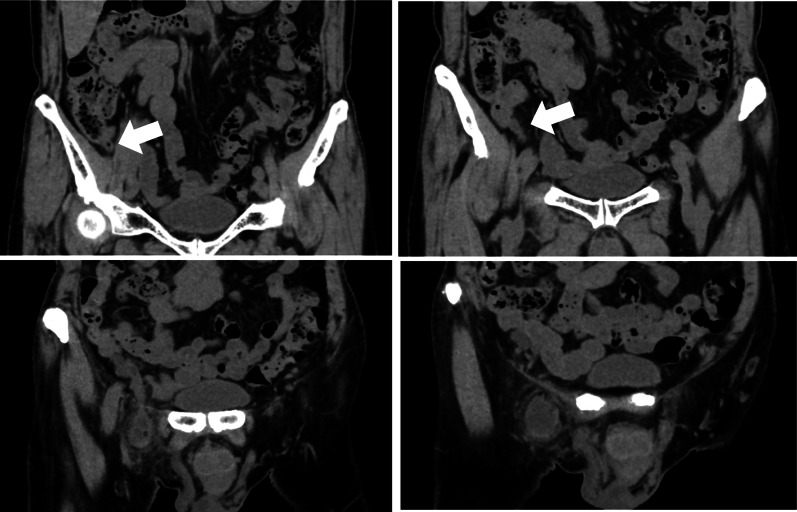
A CT scan of the abdomen shows a right femoral hernia in which the appendix is prolapsed. A mass-like accumulation of fluid was observed around the appendix in the hernia contents

The lump was located on the foot side of the inguinal ligament, but there was no discernible redness in the area. Palpation revealed that the lump was elastic and hard, with no tenderness. The patient’s abdomen was soft, also without tenderness. The white blood cell count was 5500 /µL, the C-reactive protein level was 1.06 mg/dL, and other values in the blood test were normal. Abdominal plain computed tomography (CT) revealed a right femoral hernia that contained the appendix (Fig. 1). He was diagnosed with an incarcerated appendix into a right femoral hernia and underwent surgery on the same day.

**Fig. 2:**
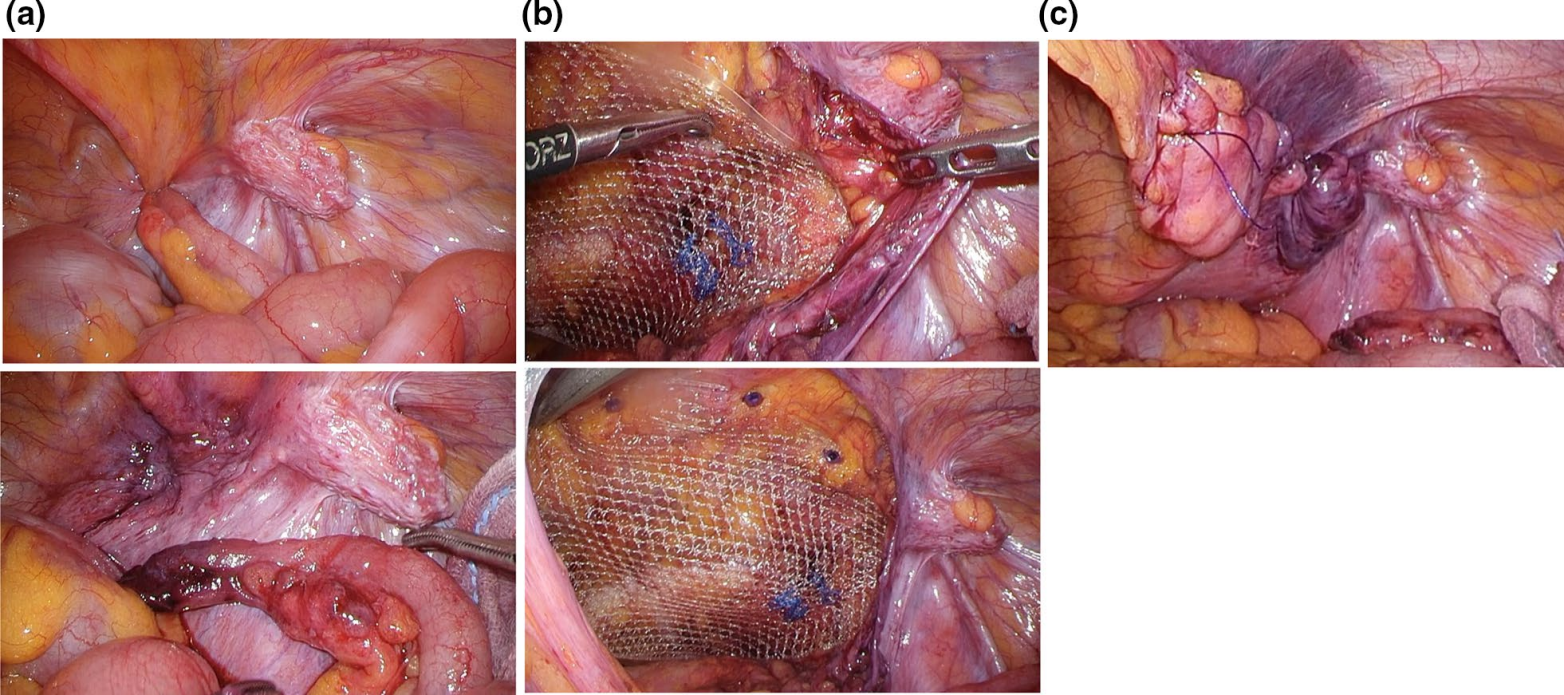
**a** Intra-abdominal findings. The mesh-plug blocking the right internal inguinal ring, and the femoral hernia where the appendix had been incarcerated was observed. **b** Deployment of new mesh. The new mesh was rotated 180 degrees and the inner edge was then inserted into the dorsal side of the existing mesh plug (outside the femoral ring). **c** The peritoneum was closed without any defects

Laparoscopic surgery was initiated by inserting a 12 mm port above the navel and 5 mm ports in the left and right lower abdomen. The mesh-plug blocking the right internal inguinal ring could be seen through the peritoneum, and the femoral hernia where the appendix had been incarcerated was visible (Fig. 2a). The appendix was relieved by traction of the incarcerated appendix itself, the appendiceal mesentery, and the peritoneum around the femoral ring. Thereafter, careful observation of the appendix and femoral ring area was performed using laparoscopy. There was no white mass observed around the appendix, and although there was serous ascites inside the hernia, there was no pus. Furthermore, although the appendix was slightly dark red, it was not clearly necrotic. The area around the internal inguinal ring where the mesh plug was placed was scarred and severely hard, making it difficult to free the peritoneum. However, the prevesical space located medial to the internal inguinal ring could be expanded without any problems, allowing sufficient exposure of Hesselbach’s triangle. The area around the femoral ring was also dissected as much as possible.

As a new mesh was deployed (3D Light Mesh; Bard, Inc, New Providence, NJ), we particularly focused on making sure that it overlapped as much as possible around the femoral ring. The new mesh was rotated 180 degrees and the inner edge was then inserted into the dorsal side of the existing mesh plug (outside of the femoral ring) (Fig. 2b). Since the inner part of the 3D mesh is designed to be wider than the outer part, we considered it would be effective to deploy the new mesh in this way to cover not only the femoral ring, but also Hesselbach’s triangle as widely as possible. The peritoneum was closed without any defects (Fig. 2c). To perform the appendectomy, a single-use ligature (2-0 polydioxanone (PDS) EndoLoop ligature; Ethicon, Cincinnati, OH) was used at the proximal end of the appendix to close the appendiceal stump.

**Fig. 3:**
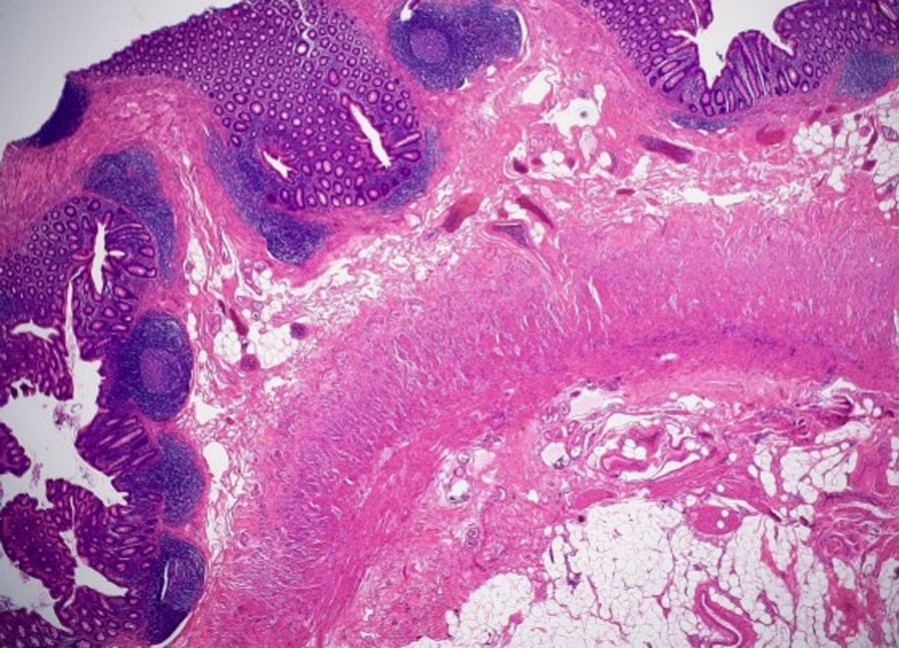
There was mild infiltration of lymphocytes and eosinophils throughout the appendiceal wall. Although no neutrophil infiltration was observed, fibrin precipitation was visible on a part of the serosal surface

Oral intake was initiated on postoperative day 2, but a fever of 38 degrees occurred in the evening of that day, and treatment with antibiotics (CMZ 2 g/day) was started for 3 days. The fever subsided the day after it started, and no fever occurred thereafter. There was also no increase in inflammation values in blood tests. He was discharged from the hospital on postoperative day 8. Pathological examination revealed no infiltration of neutrophils into the appendiceal mucosa or deeper layer, therefore no inflammation occurred (Fig. 3), and neoplasm was also not found.

## Discussion

Although we used a mesh in surgery for the De Garengeot’s hernia, the necessity of using mesh or appendectomy must be carefully determined considering the potential risk for mesh infection. Kalles’s review of 36 patients with De Garengeot’s hernia found that there were no wound infections, including no mesh infection, for either 81% of patients who underwent hernia closure with sutures or for the remaining 19% of patients who were treated with mesh [3]. In Linder’s more recent review of 87 patients with De Garengeot’s hernia, complications that occurred in 11% of cases were all related to appendicitis without any mesh infections [4]. In both reports, mesh was not used when severe inflammation was observed in and around the appendix.

According to a review of 12,402 patients with incarcerated inguinal hernias by Marcoli, mesh-based repairs reduce recurrence rates and lead to shorter hospital stays and operative times without increasing surgical site infections, mortality, seroma, chronic, plasma, and other postoperative complications [5]. However, in a subgroup analysis of patients undergoing intestinal resection, mesh repair was associated with an increased risk of surgical site infection. Papakonstantinou’s review of Amyand’s hernia cases made the following recommendations regarding the relationship between appendectomy and use of mesh [6]: if there is a perforation of the appendix or a periappendiceal abscess occurs in the inguinal canal (CDC class III/VHWG grade 2–3), synthetic mesh placement should be avoided and repair with biologic mesh or sutures should be performed. For cases with severe necrotizing inflammation or fasciitis of the inguinal canal (CDC For Class IV/VHWG Grade 4), mesh placement should be deferred to a later date and suture repair attempted if possible. The use of mesh in this case was appropriate as there was little concern about postoperative infection. Mesh-related infections can occur weeks or even years after hernia repair [7]. Furthermore, given the uncertainty in the visual diagnosis of appendiceal perforation, the use of mesh should be done with great caution, and ongoing monitoring for any potential infection is required.

Laparoscopic surgery for femoral hernias, like other groin hernias, requires exposure of the Hesselbach’s triangle, internal inguinal ring, and femoral ring, which make up the myopectineal orifice (MPO), and the MPO needs to be sufficiently covered with mesh. In this present case, the scarring around the internal inguinal ring was severe due to previous surgery with a mesh plug. It was therefore not possible to remove the mesh plug to expose the internal inguinal ring or to separate the peritoneum from the mesh plug. However, through a minimally invasive laparoscopic surgical procedure, the defective femoral ring was minimally exposed for mesh repair and could be covered with a new mesh sheet. Because we focused on covering the femoral ring with mesh as much as possible, the mesh overlap in Hesselbach’s triangle was less than 3 cm. Nevertheless, we believe that we were able to provide the best surgical treatment under these circumstances.

The International Guidelines for Groin Hernia Management recommend laparoscopic surgery for recurrence after tissue repair or the Lichtenstein procedure, and anterior treatment (with the Lichtenstein procedure) after posterior treatment with totally extraperitoneal approach (TEPP) or transabdominal preperitoneal approach (TAPP) [8]. However, if laparoscopic hernia repair is difficult due to tissue scarring, it is necessary to have the flexibility to simply confirm the intraperitoneal cavity and relieve the hernia contents, and then switch to the groin incision method. In this present case, it was necessary to confirm the presence of inflammation in the peritoneum, intraperitoneal cavity, and femoral ring, and laparoscopic surgery was useful for these examination purposes.

## Conclusions

Laparoscopic surgery is useful for visually determining the degree of inflammation of the appendix and femoral ring for de Garengeot’s hernia. If inflammation is not excessive, minimally invasive surgery using mesh is possible. Our case shows that these successful surgical results also apply to patients who previously underwent inguinal hernia surgery.

## Data Availability

Not applicable.

## Abbreviations

BMI: Body mass index
CT: Computed tomography
MPO: Myopectineal orifice
TEPP: Totally extraperitoneal approach
TAPP: Transabdominal preperitoneal approach
